# Molecular recognition of the interaction between ApoE and the TREM2 protein

**DOI:** 10.1515/tnsci-2022-0218

**Published:** 2022-04-29

**Authors:** Zhenhua Mai, Wenyan Wei, Haibin Yu, Yongze Chen, Yongxiang Wang, Yuanlin Ding

**Affiliations:** Department of Critical Care Medicine, Affiliated Hospital of Guangdong Medical University, Zhanjiang 524023, China; Department of Gerontology, Affiliated Hospital of Guangdong Medical University, Zhanjiang 524023, China; Department of Epidemiology and Health Statistics, School of Public Health, Guangdong Medical University, Dongguan 523808, China; Department of Rehabilitation Medicine, Affiliated Hospital of Guangdong Medical University, Zhanjiang 524023, China

**Keywords:** Alzheimer’s disease, ApoE, TREM2, molecular dynamics

## Abstract

Alzheimer’s disease (AD) is the most common type of dementia. The ε4 allele of the apolipoprotein E (*ApoE*) gene is the strongest known genetic risk factor for late-onset AD. Triggering receptor expressed on myeloid cells 2 (TREM2) is another important risk factor affecting the AD process after *ApoE*. Emerging evidence has identified TREM2 as a putative receptor for ApoE, raising the possibility that interactions between ApoE and TREM2 modulate the pathogenesis of AD. In this study, we performed molecular docking and molecular dynamics (MD) analyses to characterize the ApoE–TREM2 interaction and further investigated the effect of the major TREM2 disease-associated mutation (R47H) on the affinity of TREM2 for ApoE. The results indicate that the binding energy between ApoE and TREM2 occurs in an isoform-dependent manner with the following potency rank order: ApoE4 > ApoE3 > ApoE2. In addition, the R47H mutant reduced the interaction between ApoE and TREM2 protein, which may be attributed to decreased hydrogen-bonding interactions, hydrophobic interactions, and electrostatic forces between ApoE and TREM2. Our study analyzed the molecular pattern of the interactions between ApoE and TREM2 and how the variants affect these interactions based on *in silico* modeling, and the results might help to elucidate the interaction mechanism between ApoE and TREM2. Additional experimental studies will be needed to verify and explore the current findings.

## 1 Introduction

Alzheimer’s disease (AD) is the most common neurodegenerative disease and is influenced by a combination of aging, genetics, environmental influences, and lifestyle factors [1]. The ε4 allele of the apolipoprotein E (*ApoE*) gene is one of the major genetic risk factors for late-onset AD [2]. Amino acid differences at either position 112 or 158 distinguish ApoE from three subtypes, ApoE2 (Cys112, Cys158), ApoE3 (Cys112, Arg158), and ApoE4 (Arg112, Arg158) [3]. These differences significantly affect the structure and stability of the ApoE protein and its ability to bind to lipids and receptors, which is considered the structural basis by which ApoE4 accelerates the pathological process of AD [4,5]. One copy of the ε4 allele increases disease susceptibility by approximately 3-fold, and two ε4 alleles cause a 12-fold higher risk of developing AD [2]. ApoE4 is also reported to be associated with a dose-dependent decrease in age at onset [6]. Conversely, ApoE2 shows a protective effect, with decreased risk and later age at onset of AD [7]. ApoE3 is neutral and is the most common type in the population. In addition to AD, ApoE4 has been reported as a genetic risk factor for several other neurodegenerative diseases, such as frontotemporal dementia, Parkinson’s disease (PD) dementia, and Lewy body dementia [8,9]. This evidence highlights the importance of ApoE in the pathogenesis of neurodegenerative disorders. The effect of the ApoE4 isoform on AD and other neurodegenerative disorders is likely dependent on its ability to interact with lipids and its receptor-binding properties as well as its interactions with other molecules [10,11]. However, ApoE isoform-induced differences in disease risk remain to be investigated.

Accumulating evidence suggests an active role of brain innate immunity in AD pathogenesis and disease progression [12,13]. Triggering receptor expressed on myeloid cells 2 (TREM2) is an innate immunomodulatory receptor that is preferentially expressed in the membrane of microglia in the central nervous system (CNS) and is functionally required for a wide variety of cellular functions, including microglial survival, phagocytosis, and proliferation [14,15]. Genome-wide association studies identified TREM2 as one of the strongest genetic risk factors for AD, following *ApoE* [16]. The inheritance of the most common TREM2 variant, R47H (arginine to histidine at position 47), impairs ligand binding and confers a markedly increased risk for developing late-onset AD [17,18] and other neurodegenerative diseases, such as amyotrophic lateral sclerosis, frontotemporal dementia, and PD [19,20,21]. TREM2 is one of the major receptors of ApoE [22,23], the interactions between ApoE and TREM2 are important in the context of AD pathogenesis [24,25], and the TREM2–ApoE pathway has been reported to be a major regulator of the microglial functional phenotype in neurodegenerative diseases [26]. Structural and biophysical studies reported that the TREM2 R47H variant does not obviously impact the protein structure or stability but probably disrupts interactions with important ligands instead [27,28]. However, the molecular pattern of the interaction between ApoE isoforms and TREM2 protein and the effect of TREM2 AD risk mutation (e.g., R47H) on the interaction between ApoE and TREM2 remain unclear. However, the detailed interactions between ApoE and TREM2 are poorly understood.

Since ApoE and TREM2 seem to be broadly involved in neurodegeneration, there is an urgent need for further investigation of ApoE–TREM2 interactions. In this study, we performed molecular docking and molecular dynamics (MD) analyses to investigate and characterize the interactions between ApoE isoforms and TREM2. These findings may help to reveal the etiology and pathological mechanism of the risk of AD and other neurodegenerative disorders mediated by ApoE and TREM2.

## 2 Materials and methods

### 2.1 Structural model preparation and molecular docking

The wild-type (WT) crystal structure of human ApoE3 (PDB ID: 2L7B) was obtained from the Protein Data Bank (PDB). The three ApoE isoforms differ from one another only at positions 112 and 158 (ApoE2: Cys112, Cys158; ApoE3: Cys112, Arg158; ApoE4: Arg112, Arg158). Therefore, the Arg residue at 158 was mutated to Cys, and the Cys residue at 112 was mutated to Arg to obtain the initial structures of the ApoE2 and ApoE4 proteins in PyMOL 2.1. Then, the ApoE isoforms were simulated for 20 ns by MD using GROMACS software [29]. The WT crystal structure of the human TREM2 (PDB ID: 5ELI) protein was also retrieved from the PDB. The initial structure of TREM2-R47H was obtained by mutating R47 to H47 in PyMOL 2.1, and then, the Amber14 force field was utilized to optimize the protein energies. First, the 2,000-step steepest descent method optimization was performed, and then, the 2,000-step conjugate gradient method was employed to further optimize the structure, and the final model was used for the subsequent analysis. The final equilibrium structure was employed for docking with the TREM2 protein. Rosetta software is a comprehensive protein design tool for studying macromolecular structures; it includes a full suite of applications, from structure prediction to the design and remodeling of proteins and nucleic acids. Rosetta’s protein–protein docking tool mainly uses the Monte Carlo (MC) algorithm to search for conformations in full space. The whole process includes two stages: low-resolution sampling and high-resolution optimization. In the low-resolution adoption phase, Rosetta can model proteins with skeletal atoms and side-chain centroids of amino acids, use the MC algorithm for spatial searching, and apply a low-resolution potential energy function to determine whether to retain a conformation. In the high-resolution optimization stage, all the heavy atoms and polar hydrogen atoms are restored, the MC algorithm is adopted for optimization, and the side chains are inserted. The scoring function is a more complete and complex all-atom potential energy function. In this study, Rosetta’s protein–protein docking tool [30] was used to pair TREM2 with ApoE2, ApoE3, and ApoE4, and a total of 1,000 conformations were collected. The default values of other parameters were used. Finally, a reasonable docking result was selected based on the scoring function and the binding mode of TREM2 and the ApoE protein. The molecular docking result may have unreasonable atomic contacts in the space of the structure, so an energy optimization method can be used to release these forces to make the models adopt more stable structures. The Amber14 force field [31] was adopted for energy optimization, and the optimization process was divided into two steps. First, the 2,000-step steepest descent method was used to optimize the structure, and then, the 2,000-step conjugate gradient method was employed to further optimize the structure. The final result was used as the model for subsequent analysis.

### 2.2 MD simulations

MD simulations were carried out by GROMACS software [29]. An Amber99Sb all-atom force field was applied to the TIP3P water model. In the process of the MD simulation, all involved hydrogen bonds were constrained by the Lincs algorithm [32], and a 2 fs time step was employed in each simulation. Electrostatic interactions were analyzed by the particle–mesh Ewald method. The truncation value of the nonbonding interaction was set to 10 Å and updated every ten steps. The simulation temperature was controlled at 300 K by using the V-Resale temperature coupling method, and the pressure was stabilized at 1 bar by using the Parrinello–Rahman method [33]. First, the steepest descent method was employed to minimize the energy of the two systems to eliminate close contacts between atoms. Then, an NVT equilibrium simulation was performed for 100 ps at 300 K. Finally, a 100 ns MD simulation was carried out for all systems, and 5,000 conformations were saved every 20 ps. The simulation results were completed by the GROMACS-embedded program.

## 3 Results and discussion

### 3.1 Quality and binding assessment of ApoE with TREM2

**Figure 1:**
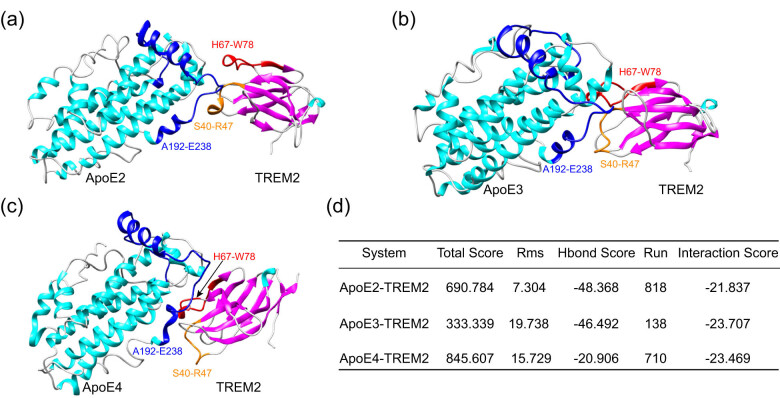
Molecular docking between ApoE genotypes and the TREM2 protein. (a) Molecular docking between ApoE2 and TREM2. (b) Molecular docking between ApoE3 and TREM2. (c) Molecular docking between ApoE4 and TREM2. (d) Interaction score between ApoE genotypes and TREM2. The blue regions represent the hinge region of ApoE, and the orange and red regions represent the CDR1 and CDR2 regions of TREM2, respectively.

In general, the accuracy of a molecular simulation depends mainly on the availability of the experimental structure and on having a good homology model for an initial condition. In three dimensions, a structure needs more than 90% of its residues in the most favored region to be a standard model of good quality and reliability. The Ramachandran plot includes three main areas: the allowed region (red), the maximum allowed region (yellow), and the disallowed region (white). The protein structure used in this study has 98% of its residues in the allowable region and no amino acid residues in the forbidden regions (Figure S1). Therefore, the structure used in this study is reliable. To analyze the binding difference between the ApoE genotypes and the TREM2 protein, the molecular docking between TREM2 and the ApoE protein was analyzed by the Rosetta program. As shown in Figure 1(a–c), the CDR1 (complementarity-determining region 1) and CDR2 regions of the TREM2 protein are important regions that interact with the ApoE protein, and the hinge region of the ApoE protein is also involved in molecular recognition between ApoE and TREM2. As shown in Figure 1d, all systems had high interaction scores (generally less than −10, indicating good binding), suggesting stable binding between ApoE and TREM2. Generally, the more amino acids participate in the interaction between two proteins, the stronger the affinity between them. TREM2 has more interaction regions (indicating a stronger affinity) with ApoE4 than those with ApoE2 and ApoE3.

### 3.2 Root-mean-square deviation (RMSD) of ApoE–TREM2 systems during MD simulations

**Figure 2:**
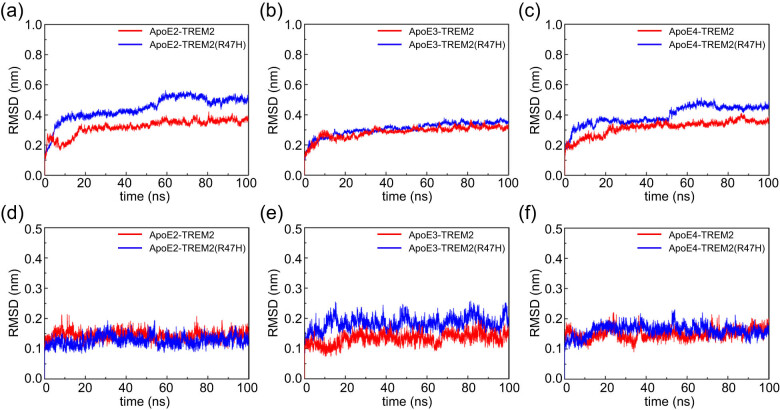
The RMSD values of the Cα atoms in the ApoE–TREM2 complex during the MD simulation. (a–c) RMSD values of the ApoE protein. (d–f) RMSD values of the TREM2 protein. The red and blue graph lines represent the ApoE–TREM2 and ApoE–TREM2 (R47H) complexes, respectively. Color scheme: red indicates ApoE–TREM2; blue indicates ApoE–TREM2 (R47H). The ordinate represents the RMSD (nm), and the abscissa represents time (ns).

The RMSD represents the average distance between backbone atoms of the superimposed proteins. It is related to the magnitude of the free motion of the atomic framework and can be used to quantify the stability of the tertiary structure of proteins. A lower RMSD value indicates that the protein system is more stable. An MD simulation analysis of 100 ns duration was carried out to further analyze the interactions of the system structure formed by ApoE and TREM2. The RMSD parameter of all systems sharply increased in the initial 20 ns (Figure 2a–c), which may be due to the strong interaction between protein and the surrounding water and solvents in the initial stage of the MD simulation, resulting in a more violent movement of the protein structure. After approximately 60 ns, the systems remained stable with minor residual fluctuations. Therefore, the trajectories from 60 to 100 ns were used for the subsequent analysis. The average RMSD values of the ApoE protein in ApoE2–TREM2 and ApoE2–TREM2 (R47H) were 0.359 and 0.510 nm and the fluctuation amplitudes were 4.06 and 4.06%, respectively (Figure 2a); in ApoE3–TREM2 and ApoE3–TREM2 (R47H), the average RMSD values were 0.313 and 0.345 nm and the fluctuation amplitudes were 4.444 and 3.484% (Figure 2b), respectively; and in ApoE4–TREM2 and ApoE4–TREM2 (R47H), the average RMSD values were 0.352 and 0.456 nm and the fluctuation amplitudes were 4.802 and 3.401%, respectively (Figure 2c). The average RMSD values of the TREM2 protein in ApoE2–TREM2 and ApoE2–TREM2 (R47H) were 0.127 and 0.141 nm, and the fluctuation amplitudes were 11.555 and 10.684%, respectively (Figure 2d); in ApoE3–TREM2 and ApoE3–TREM2 (R47H), the average RMSD values were 0.140 and 0.190 nm and the fluctuation amplitudes were 12.130 and 10.897%, respectively (Figure 2e); in ApoE4–TREM2 and ApoE4–TREM2 (R47H), the average RMSD values were 0.155 and 0.159 nm and the fluctuation amplitudes were 10.114 and 9.182%, respectively (Figure 2f). The relatively low RMSD values of ApoE and the TREM2 protein indicate that the structure of the WT system was relatively stable during the simulation process. In the mutant system, the RMSD value of the ApoE protein changed greatly, indicating that the TREM2-R47H mutation may impact the mobility of ApoE and TREM2 proteins. In addition, the RMSD value of the TREM2 protein was low, but the fluctuation was obvious during the simulation, possibly because the structure of the TREM2 protein is smaller than that of ApoE, and there is no alpha helix in the TREM2 structure, resulting in greater flexibility of the TREM2 protein.

### 3.3 Root-mean-square fluctuation (RMSF) analysis of ApoE–TREM2 systems during MD simulations

**Figure 3:**
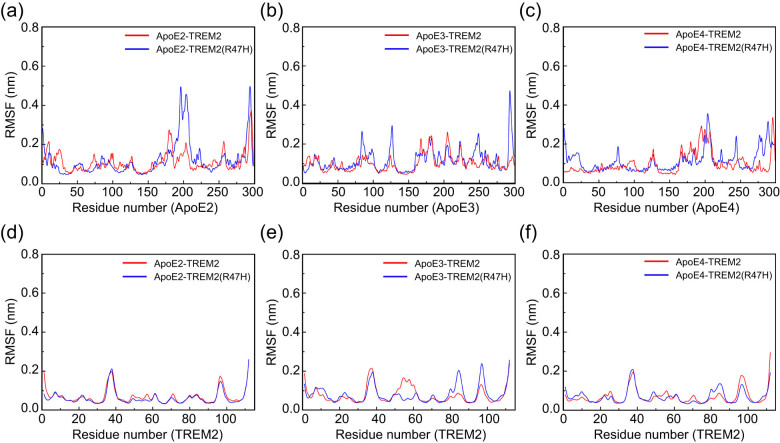
Average RMSF plots of the Cα atoms in the ApoE–TREM2 complex during the MD simulation. (a–c) RMSF plots of the ApoE protein. (d–f) RMSF plots of the TREM2 protein. The red and blue graph lines represent the ApoE–TREM2 and ApoE–TREM2 (R47H) complexes, respectively. Color scheme: red indicates ApoE–TREM2; blue indicates ApoE–TREM2 (R47H). The ordinate represents the RMSF (nm), and the abscissa represents the residue number.

The flexibility of the ApoE–TREM2 complexes was further evaluated by the RMSF value, which represents the conformational flexibility of amino acid residues. Residues with low RMSF values are more stable due to the limited movements during simulations. Overall, we observed a relatively large RMSF fluctuation of the ApoE protein during the simulation, while the flexibility of the TREM2 protein was relatively small. Specifically, in the ApoE–TREM2 systems, during simulations, the different ApoE isoforms showed greater flexibility fluctuations in some regions of the ApoE protein, and the R47H variant displayed clearly enhanced flexibility in these regions (Figure 3a–c), suggesting that the R47H variant may lead to greater structural fluctuations in part of the ApoE protein, which in turn may affect the binding of ApoE to TREM2. The R47H variant exhibited obvious changes in the flexibility distribution of the ApoE3–TREM2 complex in some regions of TREM2, suggesting that the variant may affect the binding between ApoE3 and TREM2 (Figure 3e). However, the flexibility plots of the ApoE2–TREM2 and ApoE4–TREM2 complexes were similar after R47H mutation (Figure 3d and f), suggesting that the R47H variant has little effect on the binding between ApoE2–TREM2 and ApoE4–TREM2.

### 3.4 The radius of gyration (Rg) values of the ApoE–TREM2 complexes

**Figure 4:**
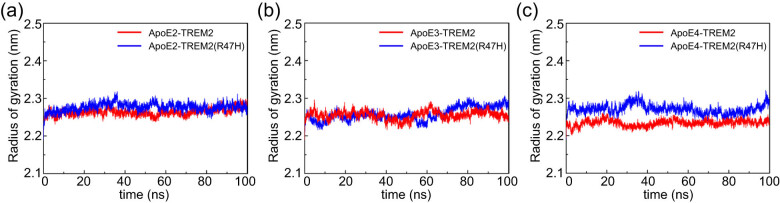
The Rg values of the ApoE–TREM2 complexes during 100 ns of MD simulation. (a) Rg values of ApoE2–TREM2 and ApoE2–TREM2 (R47H). (b) Rg values of ApoE3–TREM2 and ApoE3–TREM2 (R47H). (c) Rg values of ApoE4–TREM2 and ApoE4–TREM2 (R47H). Color scheme: red indicates ApoE–TREM2; blue indicates ApoE–TREM2 (R47H). The ordinate represents Rg (nm), and the abscissa represents time (ns).

The Rg simulation trajectories depict the volumetric oscillations of atoms in the system from their mean center of mass [34]. Rg is an important parameter reflecting protein structure compactness. To further characterize the effects of the ApoE genotypes and TREM2-R47H mutation on compactness changes in the ApoE–TREM2 complexes, the Rg of each ApoE–TREM2 system was evaluated. The Rg values of the ApoE2–TREM2 and ApoE2–TREM2 (R47H) systems were 2.268 and 2.277 nm, respectively (Figure 4a); those of the ApoE3–TREM2 and ApoE3–TREM2 (R47H) systems were 2.258 and 2.273 nm, respectively (Figure 4b); and those of the ApoE4–TREM2 and ApoE4–TREM2 (R47H) systems were 2.235 and 2.266 nm, respectively (Figure 4c). The Rg values of ApoE2 and ApoE3 with the TREM2 and TREM2 (R47H) systems did not change significantly during the simulation process, while the Rg value of the ApoE4–TREM2 complex was lower than that of ApoE4–TREM2 (R47H), indicating that the complex structures of ApoE4–TREM2 (R47H) are more expanded than those of ApoE4–TREM2; thus, it is speculated that the mutation decreased the protein interaction.

### 3.5 Changes in the hydrogen bonding between ApoE and TREM2 during MD simulations

**Figure 5:**
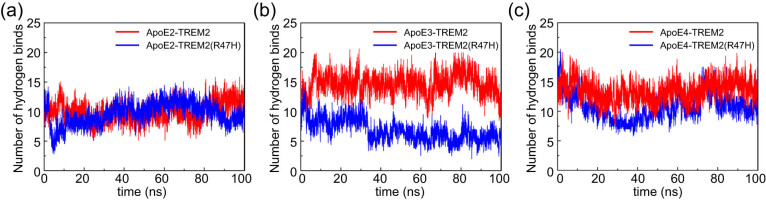
Changes in the number of hydrogen bonds between the ApoE protein and TREM2 during the MD simulation. (a) Hydrogen bond number between ApoE2 and TREM2. (b) Hydrogen bond number between ApoE2 and TREM2. (c) Hydrogen bond number between ApoE2 and TREM2. Color scheme: red indicates ApoE–TREM2, and blue indicates ApoE–TREM2 (R47H). The ordinate represents the number of hydrogen bonds, and the abscissa represents time (ns).

Hydrogen bonds are an important form of protein interaction and an important force contributing to the structural stability of protein–ligand complexes. Therefore, the hydrogen bonds that ApoE formed with the TREM2 protein at distances less than 3.5 Å (0.35 nm) and angles between the hydrogen bond donor and acceptor greater than 130° during the MD simulation were statistically analyzed. As shown in Figure 4, the average numbers of hydrogen bonds in the ApoE2–TREM2 and ApoE2–TREM2 (R47H) complexes were 10.377 and 10.632, respectively (Figure 5a); the average numbers of hydrogen bonds in the ApoE3–TREM2 and ApoE3–TREM2 (R47H) complexes were 14.760 and 5.641, respectively (Figure 5b); and the average numbers of hydrogen bonds in the ApoE4–TREM2 and ApoE4–TREM2 (R47H) complexes were 13.964 and 11.798, respectively (Figure 5c). The average number of hydrogen bonds between ApoE2 and TREM2 was lower than the number of hydrogen bonds between ApoE3 and TREM2 and between ApoE4 and TREM2. In addition, the average number of hydrogen bonds between ApoE3 and TREM2 was obviously higher than that in the ApoE3–TREM2 (R47H) complex, indicating that the R47H variants decreased the stability of the ApoE3–TREM2 complex.

### 3.6 The differences in residue interactions between ApoE isoforms and the TREM2 protein

**Figure 6:**
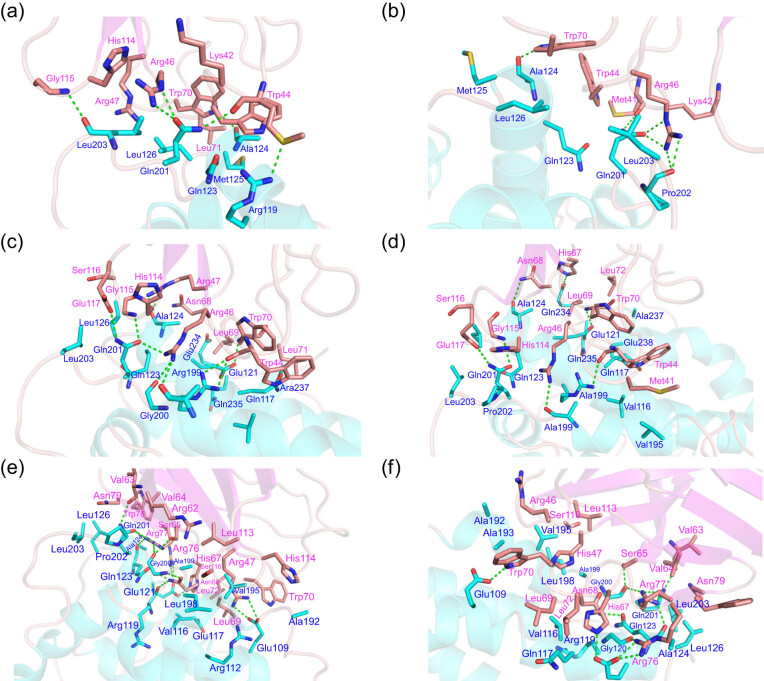
Three-dimensional binding mode between ApoE and the TREM2 protein. (a and b) The three-dimensional binding mode of ApoE2–TREM2 and ApoE2–TREM2 (R47H). (c and d) The three-dimensional binding mode of ApoE3–TREM2 and ApoE3–TREM2 (R47H). (e and f) The three-dimensional binding mode of ApoE4–TREM2 and ApoE4–TREM2 (R47H). The green dashed line indicates the hydrogen bond, and blue and purple indicate the amino acid residues in the ApoE and TREM2 proteins.

To obtain further information about the amino acid residues and the interactions between ApoE and TREM2, the binding mode of ApoE and TREM2 was analyzed after MD simulations. The amino acid residues involved in the molecular recognition of ApoE and TREM2 are summarized in Table 1. There were six hydrogen bonds in ApoE2–TREM2 and ApoE2–TREM2 (R47H) (Figure 6a and b, Figure S2a and b). Hydrogen bonds present in both systems, namely, Trp70···Ala124 and Arg46···Glu201, were identified as important contributions to the binding of ApoE2 with TREM2. In addition, there are some hydrophobic amino acids at the binding interface of ApoE2 and TREM2, such as Ala124, Leu126, Met41, and Trp70, that further enhance the affinity of the proteins. When Arg47 was mutated to His47, due to the shorter side chain and stronger rigidity, His47 did not participate in the molecular recognition of ApoE2 and TREM2. Therefore, it can be speculated that the R47H mutation reduces the binding force between ApoE2 and TREM2. As shown in Figure 6c and d and Figure S2c and d, ApoE3–TREM2 and ApoE3–TREM2 (R47H) formed 11 and 6 hydrogen bonds, respectively. The same number of hydrogen bonds in the two systems existed between Trp40···Arg119, Arg46···Ala199, and Trp70···Glu121, indicating that these hydrogen bonds are important in ApoE3–TREM2 and ApoE3–TREM2 (R47H). In addition, there are some hydrophobic amino acids at the binding interface of ApoE3 and TREM2, such as Val116, Ala124, Leu126, Ala199, Leu203, Ala237, Trp44, Trp70, and Leu71. The hydrophobic effect formed by these amino acid residues further enhances the affinity of the proteins. When Arg47 was mutated to His47, the hydrogen bond between Arg47 and Ala124 disappeared due to the change in polarity and His47 did not participate in the protein interaction. Therefore, it can be speculated that the R47H mutation decreases the binding force of ApoE3 and TREM2. As shown in Figure 6e and f and Figure S2e and f, ApoE4–TREM2 and ApoE4–TREM2 (R47H) formed 14 and 11 hydrogen bonds, respectively. The same number of hydrogen bonds formed between Val63···Gln201, Ser6···Gly200, His67···Glu201, Asn68···Gln123, Arg76···Glu121, and Arg77···Gln201, indicating that these hydrogen bonds are important in ApoE4 binding to TREM2. In addition, there are some hydrophobic amino acids at the binding interface of ApoE4 and TREM2, such as Val116, Ala124, Leu126, Ala192, Val195, Leu198, Ala199, Leu203, Val63, Val64, Leu69, Trp70, Leu72, and Leu113. The hydrophobic interaction formed by the residues can further enhance the affinity of ApoE4 and TREM2. When Arg47 was mutated to His47, the hydrogen bond and electrostatic interaction between Arg47 and Glu109 disappeared due to the changes in polarity; as a result, the interaction between His47 and ApoE4 relied only on hydrophobic interactions, so it can be speculated that the R47H mutation significantly decreases the binding force of ApoE4 and TREM2.

**Table 1:** Amino acid residues involved in the molecular recognition of ApoE and TREM2

System	Interaction residues in ApoE	Interaction residues in TREM2
ApoE2–TREM2	Arg119, Gln123, Ala124, Met125, Leu126, Gln201, Leu203	Met41, Lys42, Trp44, Arg46, Arg47, Trp70, His114
ApoE2–TREM2 (R47H)	Gln123, Ala124, Met125, Leu126, Gln201, Pro202, Leu203	Met41, Lys42, Trp44, Gly45, Arg46, Trp70
ApoE3–TREM2	Val116, Gln117, Arg119, Glu121, Gln123, Ala124, Leu126, Ala199, Gly200, Gln201, Leu203, Gln235, Ala237	Trp44, Arg46, Arg47, Asn68, Trp70, Leu71, His114, Gly115, Ser116, Glu117
APOE3–TREM2 (R47H)	Val116, Gln117, Arg119, Glu121, Gln123, Ala124, Val195, Ala199, Gln201, Pro202, Leu203, Glu234, Gln235, Ala237, Glu238	Met41, Trp44, Arg46, His67, Asn68, Leu69, Trp70, His114, Gly115, Ser116, Glu117
APOE4–TREM2	Glu109, Arg112, Val116, Gln117, Arg119, Glu121, Gln123, Ala124, Leu126, Ala192, Val195, Leu198, Ala199, Gly200, Gln201, Pro202, Leu203	Arg47, Arg62, Val63, Val64, Ser65, His67, Asn68, Leu69, Trp70, Leu72, Arg76, Arg77, Trp78, Asn79, Leu113, His114, Ser116,
APOE4–TREM2 (R47H)	Glu109, Val116, Gln117, Arg119, Gly120, Glu121, Gln123, Ala124, Leu126, Ala192, Ala193, Val195, Leu198, Ala199, Gly200, Gln201, Leu203,	Arg46, His47, Val63, Val64, Ser65, Asn68, Leu69, Trp70, Leu72, Arg77, Trp78, Asn79, Leu113, Ser116

### 3.7 Binding energy between the ApoE protein and TREM2

**Figure 7:**
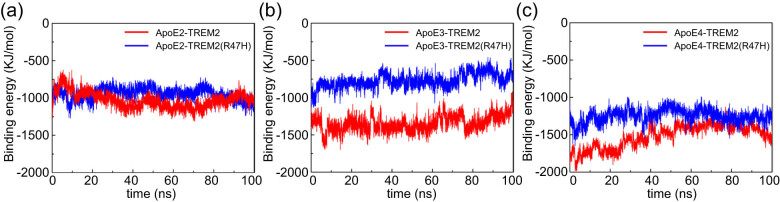
Changes in the binding energy between the ApoE isoform and TREM2 during the MD simulation. (a) Binding energies of ApoE2–TREM2 and ApoE2–TREM2 (R47H). (b) Binding energies of ApoE3–TREM2 and ApoE3–TREM2 (R47H). (c) Binding energies of ApoE4–TREM2 and ApoE4–TREM2 (R47H). Color scheme: red indicates ApoE–TREM, and blue indicates ApoE–TREM (R47H). The ordinate represents the binding energy (kJ/mol), and the abscissa represents time (ns).

The different binding modes lead to differences in binding energy; therefore, the binding energy between ApoE and TREM2 was further analyzed. As shown in Figure 6, the binding energies of the six systems were basically stable after approximately 60 ns of simulations. The average binding energies of the ApoE2–TREM2 and ApoE2–TREM2 (R47H) systems were −1066.48 and −970.50 kJ/mol, respectively (Figure 7a). The average binding energies of the ApoE3–TREM2 and ApoE3–TREM2 (R47H) systems were −1284.70 and −720.81 kJ/mol, respectively (Figure 7b). The average binding energies of the ApoE4–TREM2 and ApoE4–TREM2 (R47H) systems were −1405.13 and −1250.11 kJ/mol, respectively (Figure 7c). These results indicate that the binding energy between ApoE and TREM2 was isoform-dependent, with ApoE4 > ApoE3 > ApoE2. Judging from the binding energy data, the R47H variant had a clearly decreased binding affinity to ApoE3. However, the binding affinities of ApoE2 and ApoE4 seemed to be only slightly altered. These results are consistent with the RMSF values, which indicate that R47H tends to affect the binding between ApoE3 and TREM2 but has only a slight effect on the binding affinities between ApoE2–TREM2 and ApoE4–TREM2. In addition, our study is consistent with previous investigations reporting that the R47H mutant reduced the binding energy between ApoE and TREM2. Kober et al. evaluated the binding between TREM2 and nonlipidated ApoE based on biolayer interferometry (BLI) analysis and reported that the R47H variant disrupted the binding affinity [35]. Moreover, the authors found that the major hinge region of ApoE (mainly residues 192–238) is most likely responsible for TREM2 binding. Consistently, our study reported that the hinge region of the ApoE protein is important for the molecular recognition between ApoE and TREM2. Notably, Jendresen et al. reported that amino acids 130–149 in the N-terminal domain of the ApoE protein are also involved in TREM2 binding [36]. In addition, by using several different techniques, such as dot-blot binding assays, solid-phase binding methods, and enzyme-linked immunosorbent assay (ELISA)-based binding experiments, researchers confirmed that the R47H variant reduced TREM2 affinity to bind ApoE [23,37]. However, pull-down and BLI assay studies also showed that the R47H variant did not significantly affect the binding between sTREM2 and lapidated ApoE3, while the variant disrupted sTREM2 binding to lapidated ApoE4 [38]. Inconsistent results may be attributable to the manner of TREM2 presentation and the different detection techniques employed in these studies, as some studies used Fc-TREM2 fusion protein dimers, which may influence protein avidity and biphasic binding, and some use monomeric TREM2 or sTREM2 ectodomains to detect interactions. Notably, we further found that the R47H mutant decreased the interaction between ApoE and the TREM2 protein, which may be attributed to the reduced hydrogen-bonding interactions, hydrophobic interactions, and electrostatic forces between them. For ApoE isoforms, our results observed that ApoE binds to the TREM2 receptor with greater stability and rigidity with a potency order ApoE4 > ApoE3 > ApoE2, which is consistent with a previous study that reported slight affinity differences between isoforms and TREM2 by using a BLI assay [36]. The higher binding for the ApoE4 isoform might lead to more potent TREM2 signaling and the potential for higher microglial activation; in this scenario, ApoE4 contributes to the onset risk for AD. In contrast, no genotype differences were detected between ApoE isoforms and TREM2 in most previous studies [23,36,37]. The effect of the TREM2–R47H variant and ApoE isoforms on the interaction between ApoE and TREM2 is summarized in Table S1. Differential binding affinities may be due to the lipidation state of ApoE; although ApoE lipidation may not be necessary for TREM2 binding, lipidation appeared to influence the interaction between ApoE and TREM2 [35], and ApoE isoforms were reported to have differential capacity to bind to lipids [39].

## 4 Conclusion

In this study, molecular docking and MD simulation analyses were used to study the molecular recognition of ApoE isoforms and TREM2 proteins and the effect of the TREM2-R47H mutation on these interactions. Protein docking results showed that the hinge region of the ApoE protein and the CDR1 and CDR2 regions of the TREM2 protein were the main participants in molecular recognition. Furthermore, based on the RMSD, Rg, binding energy, and hydrogen bond results for the amino acid residues that differed between the ApoE isoforms and the TREM2 protein, we found that ApoE binds to the TREM2 receptor with greater stability and rigidity in the order ApoE4 > ApoE3 > ApoE2, and the R47H mutation disrupts the binding of ApoE and TREM2. In addition to previous findings, this study provides valuable new insights into how the R47H mutation decreases the interaction between ApoE and TREM2. In summary, our study, along with previously published data, will help to reveal the etiology and pathologic mechanism of the risk of AD and other neurodegenerative disorders mediated by ApoE and TREM2. While the results are based on *in silico* modeling, further structural and biophysical experiments, such as X-ray crystallography, may provide detailed mechanistic insights into the binding interactions of TREM2 and ApoE.

## Supplementary Material

Supplementary Figure
