# Supplementary material for: Molecular recognition of the interaction between ApoE and the TREM2 protein
Source: Transl Neurosci. 2022 Apr 29;13(1):93–103. doi: 10.1515/tnsci-2022-0218 (PMC9055258; doi:10.1515/tnsci-2022-0218)
Supplement: Supplementary Figure [file tnsci-2022-0218-sm.pdf]

Supplementary material

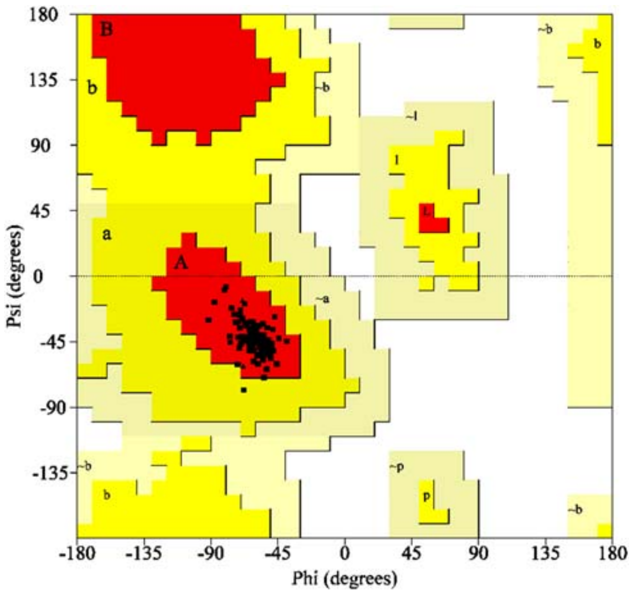

Figure S1: Ramachandran plot of ApoE protein. The Ramachandran plot includes three main areas: the allowed region (red), the maximum allowed region (yellow), and the disallowed region (white).

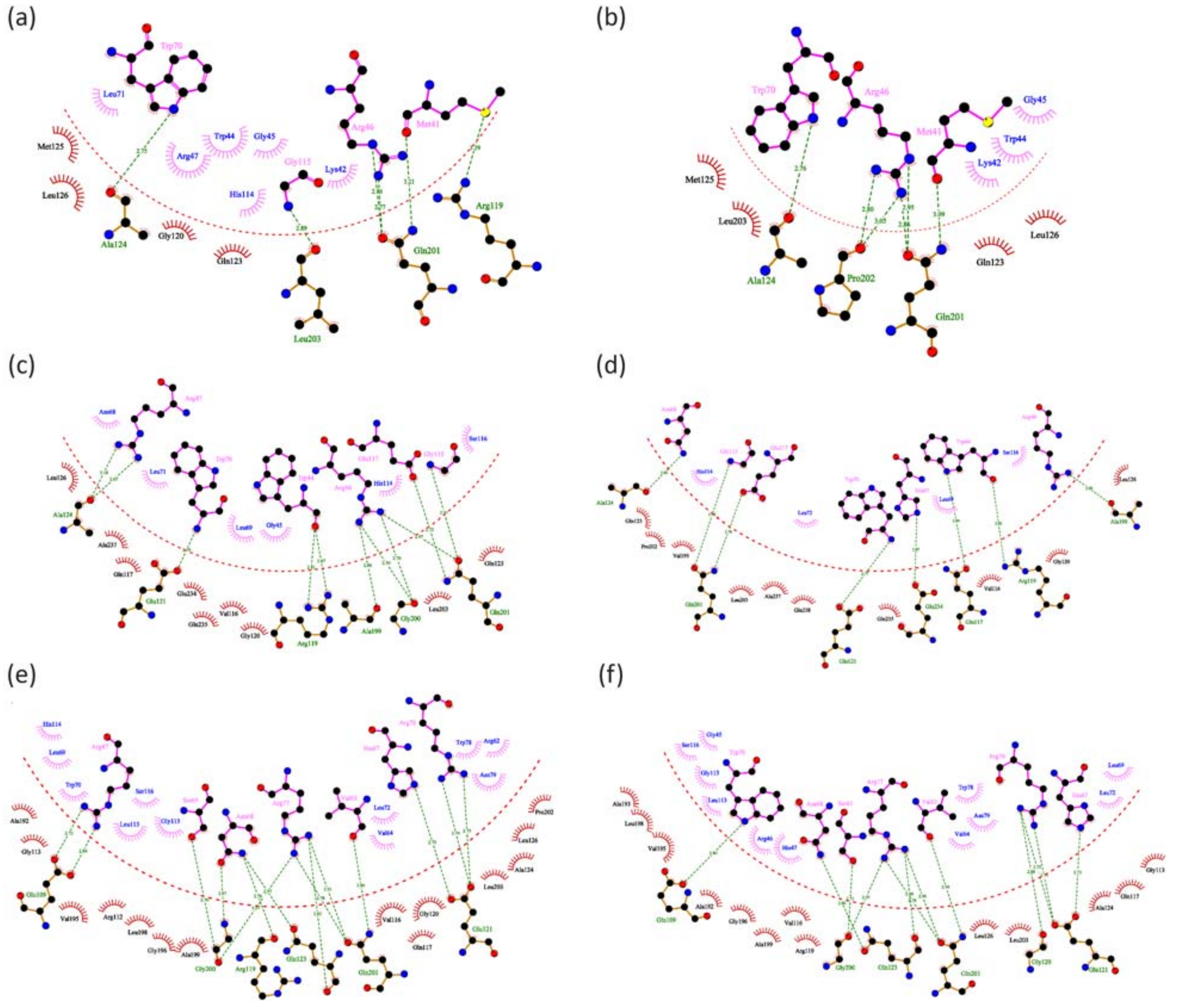

Figure S2: Two-dimensional binding pattern modes between ApoE and TREM2. (a and b) The two-dimensional binding mode of ApoE2-TREM2 and ApoE2-TREM2 (R47H). (c and d) The two-dimensional binding mode of ApoE3-TREM2 and ApoE3-TREM2 (R47H). (e and f) The two-dimensional binding mode of ApoE4-TREM2 and ApoE4-TREM2 (R47H). The green dashed lines indicate hydrogen bonds, the red dashed lines indicate the combined interface, and blue and purple indicate the amino acid residues in ApoE and TREM2, respectively. Red gears and purple gears indicate hydrophobic interactions.

Table S1: Interactions between APOE and TREM2

Interactions between APOE and TREM2	Methods	Results	Reference
Nonlipidated APOE2, APOE3, APOE4 with TREM2	BLI	E4 > E3 > E2	[1]
Nonlipidated APOE2, APOE3, APOE4 with sTREM2	BLI	No significant difference	[1]
TREM2 and TREM2 (R47H) with APOE	BLI	R47H reduced binding	[1]
Nonlipidated APOE2, APOE3, APOE4 with TREM2	ELISA	No significant difference	[2]
Lapidated APOE2, APOE3, APOE4 with TREM2	Dot-blot binding assay	No significant difference	[3]
TREM2 and TREM2 (R47H) with APOE	Dot-blot binding assay and solid-phase binding assay	R47H reduced binding	[3]
APOE2, APOE3, APOE4 with TREM2	ELISA	No significant difference	[4]
TREM2 and TREM2 (R47H) with APOE	ELISA	R47H reduced binding	[4]
TREM2 and TREM2 (R47H) with lapidated APOE	BLI	R47H reduced binding	[5]
sTREM2 and sTREM2 (R47H) with lapidated APOE3	Pull-down assay and BLI	No significant difference	[6]
sTREM2 and sTREM2 (R47H) with lapidated APOE4	BLI	R47H increased binding	[6]

BLI: biolayer interferometry; ELISA: enzyme-linked immunosorbent assay.

References

[1] Kober DL, Stuchell-Brereton MD, Kluender CE, Dean HB, Strickland MR, Steinberg DF, et al. Functional insights from biophysical study of TREM2 interactions with apoE and Abeta1-42. Alzheimers Dement. 2020
[2] Jendresen C, Arskog V, Daws MR, Nilsson LN. The Alzheimer's disease risk factors apolipoprotein E and TREM2 are linked in a receptor signaling pathway. J Neuroinflammation. 2017;14(1):59.
[3] Atagi Y, Liu CC, Painter MM, Chen XF, Verbeeck C, Zheng H, et al. Apolipoprotein E is a ligand for triggering receptor expressed on myeloid cells 2 (TREM2). J Biol Chem. 2015;290(43):26043–50.
[4] Bailey CC, DeVaux LB, Farzan M. The triggering receptor expressed on myeloid cells 2 binds Apolipoprotein E. J Biol Chem. 2015;290(43):26033–42.
[5] Yeh FL, Wang Y, Tom I, Gonzalez LC, Sheng M. TREM2 Binds to apolipoproteins, including APOE and CLU/APOJ, and thereby facilitates uptake of amyloid-beta by microglia. Neuron. 2016;91(2):328–40.
[6] Lessard CB, Malnik SL, Zhou Y, Ladd TB, Cruz PE, Ran Y, et al. High-affinity interactions and signal transduction between Abeta oligomers and TREM2. EMBO Mol Med. 2018;10:11.
